# Declines in Representational Quality and Strategic Retrieval Processes Contribute to Age-Related Increases in False Recognition

**DOI:** 10.1037/xlm0000412

**Published:** 2017-05-22

**Authors:** Alexandra N. Trelle, Richard N. Henson, Deborah A. E. Green, Jon S. Simons

**Affiliations:** 1 Department of Psychology, University of Cambridge, and Behavioural and Clinical Neuroscience Institute, Cambridge, United Kingdom; 2 MRC Cognition and Brain Sciences Unit, Cambridge, United Kingdom; 3 Department of Psychology, University of Cambridge, and Behavioural and Clinical Neuroscience Institute, Cambridge, United Kingdom

**Keywords:** aging, false recognition, interference, representational quality, strategic retrieval

## Abstract

In a Yes/No object recognition memory test with similar lures, older adults typically exhibit elevated rates of false recognition. However, the contributions of impaired retrieval, relative to reduced availability of target details, are difficult to disentangle using such a test. The present investigation sought to decouple these factors by comparing performance on a Yes/No (YN) test to that on a Forced Choice (FC) test, which minimizes demands on strategic retrieval processes, enabling a more direct measure of the availability of object details. Older adults exhibited increased lure false recognition across test formats (Experiment 1), suggesting a decline in the availability of object details contributes to deficits in performance. Manipulating interference by varying the number of objects studied selectively enhanced performance in the FC test, resulting in matched performance across groups, whereas age differences in YN performance persisted (Experiment 2), indicating an additional contribution of impaired strategic retrieval. Consistent with differential sensitivity of test format to strategic retrieval and the quality of stimulus representations among older adults, variability in the quality of object representations, measured using a perceptual discrimination task, was selectively related to FC performance. In contrast, variability in memory control processes, as measured with tests of recall and executive function, was related to performance across test formats. These results suggest that both declines in the availability of object details and impaired retrieval of object details contribute to elevated rates of lure false recognition with age, and highlight the utility of test format for dissociating these factors in memory-impaired populations.

The episodic memory deficits experienced by older adults are characterized not only by increased forgetting, but also greater susceptibility to false memories of events that did not occur (see Devitt & Schacter, 2016 for review). One particularly robust example of false memory among older adults is the false recognition of novel objects that are perceptually similar to studied objects in a recognition memory test. Despite this increased tendency to incorrectly identify similar foils as having been studied previously, the ability to correctly identify previously studied targets as old, and identify novel and perceptually distinct foils as new, is typically unaffected (Koutstaal, 2003; Toner, Pirogovsky, Kirwan, & Gilbert, 2009; Yassa et al., 2011; Holden, Toner, Pirogovsky, Kirwan, & Gilbert, 2013). This deficit has proven to be resistant to a number of task manipulations, including those that aim to enhance attention to perceptual detail during encoding and those that encourage more strict responding during retrieval (Koutstaal, Schacter, Galluccio, & Stofer, 1999; Stark, Stevenson, Wu, Rutledge, & Stark, 2015). Despite the frequent emergence and stability of this pattern, our current understanding of the basis for elevated rates of false recognition of lures among older adults remains limited.

One reason that a Yes/No recognition test with similar lures reveals greater age-related effects than do typical tests of item recognition may be related to the increased demands it places on recollection-based retrieval strategies (Migo, Montaldi, Norman, Quamme, & Mayes, 2009; Norman, 2010). In particular, because targets and foils are perceptually similar and therefore both highly familiar, it is difficult to reliably distinguish targets from lures using a strength-based criterion alone. Instead, participants must use a recollection-based retrieval strategy (i.e., recall-to-reject) to support performance (Migo et al., 2009). This strategy describes the process of disqualifying an exemplar as having been studied by first recalling details of the studied target, and then detecting a mismatch between the target and the lure (Brainerd, Reyna, Wright, & Mojardin, 2003; Gallo, 2004). Critically, the ability to use this strategy successfully requires both that sufficient information about the target is available to disqualify the lure as having been studied, as well as the ability to selectively retrieve and evaluate stored details about the target. Thus, age-related increases in false recognition could plausibly arise due to declines in either of these factors, or a combination of the two.

The relative contribution of each of these factors to elevated rates of lure false recognition among older adults remains unclear. On the one hand, existing evidence suggests that aging negatively affects the ability the implement controlled and strategic retrieval processes, relative to more spontaneous and automatic processes (Jennings & Jacoby, 1993; Yonelinas, 2002). For example, older adults typically exhibit disproportionate deficits in memory performance under conditions that place similar demands on recollection-based retrieval strategies, such as rejecting recombined pairs in an associative recognition test (Castel & Craik, 2003; Cohn, Emrich, & Moscovitch, 2008), or rejecting studied items from a nontarget source in an exclusion paradigm (Gallo, Bell, Beier, & Schacter, 2006; Jennings & Jacoby, 1993). Conversely, age differences are typically absent when performance can be supported based on the presence or absence of item familiarity, such as endorsing studied pairs and rejecting experimentally novel foils (Cohn et al., 2008; Gallo et al., 2006; Yonelinas, 2002). As the ability to strategically retrieve and evaluate stored details appears to be impaired with age, this factor alone could account for elevated rates of false recognition among older adults.

However, it could also be the case that aging is associated with declines in the availability of object details that are necessary to disqualify lures as having been studied. Evidence for this possibility has been mixed. Previous work has found that despite impaired explicit memory for object details, implicit memory remained intact with age (Koutstaal, 2003), suggesting that object details may be available even when they are not retrieved successfully. In contrast, other work has identified age-related impairments in perceptual discrimination tasks that require participants to distinguish between stimuli sharing overlapping features, even though such tasks minimize mnemonic demands (Lee, Smith, Grady, Hoang, & Moscovitch, 2014; Ryan et al., 2012; Yeung, Ryan, Cowell, & Barense, 2013). Notably, if the availability of object details is reduced with age, this could also be the sole factor driving age-related increases in false recognition. That is, if object details that can disambiguate targets and foils are not available, a mismatch between targets and foils cannot be successfully detected, rendering a recall-to-reject strategy unsuccessful.

Critically, in a typical Yes/No object recognition test with similar foils, the availability of object details and the ability to retrieve and evaluate these details are confounded, making it difficult to tease apart the relative contributions of these two factors to false recognition. One way of overcoming this limitation is to hold demands on the availability of object details constant, while varying demands on strategic retrieval processes. Existing empirical and modeling evidence indicates that this can be done by comparing performance in a typical Yes/No recognition memory format to performance in a Forced Choice test format, wherein targets and corresponding foils are presented simultaneously (Guerin, Robbins, Gilmore, & Schacter, 2012; Holdstock et al., 2002; Migo et al., 2009, 2014; Norman, 2010). In particular, whereas the item-wise presentation of targets and foils in the Yes/No test places considerable demands on recollection-based processes (i.e., recall-to-reject), the simultaneous presentation of targets and their corresponding foils in a Forced Choice test can be supported by judgments of relative familiarity differences between the two choices (Norman, 2010).

In support of this proposal, behavioral work in younger adults has found that high rates of lure false recognition in a Yes/No test can be reduced substantially by presenting targets and corresponding foils simultaneously at test (Guerin et al., 2012). This pattern suggests that the presentation of corresponding foils increases the accessibility of stored details, likely by reducing demands on strategic retrieval processes. Consistent with this possibility, previous work in younger adults using a modified Remember/Know procedure has found that successful performance in the Yes/No test format is supported predominantly by the use of a ‘recall-to-reject’ strategy, whereas accurate performance in the Forced Choice format can be supported by relying on familiarity alone (Migo et al., 2009). Similarly, previous work in older adults has identified distinct relationships between independent standardized measures of recall and recognition memory, and performance in the Yes/No and Forced Choice tests, respectively (Migo et al., 2014).

Collectively, these observations indicate that the simultaneous presentation of targets and foils in the Forced Choice test considerably reduces demands on strategic retrieval, thereby enabling a more direct assessment of the availability of object details and the contribution of this factor to increases in false recognition. In the present study, we directly compare older and younger adults’ performance across test formats to characterize the relative contribution of these two factors to elevated rates of lure false recognition with age. If older adults exhibit selective deficits in Yes/No performance, coupled with intact Forced Choice performance, then this would suggest that age-related increases in false recognition are driven primarily by impairments in strategic retrieval processes. However, if older adults also exhibit deficits in Forced Choice performance, this would suggest that a decline in the availability of object details that are necessary to disambiguate targets and foils contributes to increases in lure false recognition with age, and may be the primary factor driving impaired performance.

## Experiment 1

The primary aim of this experiment was to investigate the degree to which age differences in Yes/No recognition memory with similar foils are ameliorated when demands on recall-to-reject are reduced through the simultaneous presentation of targets and corresponding foils at test. To this end, we compared older and younger adults’ recognition memory performance across Yes/No and Forced Choice test formats. If age-related increases in false recognition in the Yes/No test arise solely as a result of impairments in the strategic retrieval and evaluation of item information, we should observe deficits in Yes/No performance coupled with intact Forced Choice performance. In contrast, age differences in Forced Choice performance would suggest that a reduction in the availability of disambiguating object details contributes to false memory errors among older adults.

A second aim of this experiment was to gain further support for the proposal that successful performance in the Yes/No test and Forced Choice test differentially relies on the use of recollection and familiarity. To this end, we included the modified Remember-Know (RK) judgments that were used in previous work (Migo et al., 2009) to provide an indication of the strategy participants use to make their decisions. Specifically, participants were asked to provide a ‘remember’ response if their decision was based on retrieval of specific target details, indicating the use of a recall-to-reject or recall-to-accept strategy, and a ‘familiar’ response if their decision was based on the relative familiarity of the presented exemplar, in the absence of retrieval of specific stimulus details. We predicted that performance in the Yes/No test would rely primarily on the successful use of a recall-to-reject strategy, whereas performance in the Forced Choice test could be more successfully supported by familiarity-based judgments, as described in previous work (Migo et al., 2009). Moreover, we predicted that age differences in the ability to successfully execute a recollection-based retrieval strategy would be greater than age differences in accurate familiarity-based responding (Koen & Yonelinas, 2016).

### Method

#### Participants

Thirty-two younger adults aged 18 to 28 years (*M* = 22.66, *SD* = 3.04) and 32 older adults aged 60–80 years (*M* = 70.47, *SD* = 4.59) participated. All participants were native English speakers. The younger adults were students from the University of Cambridge and the older adults were healthy, community-dwelling volunteers. The groups were matched with respect to years of formal education (*t* < 1) and the older adults outperformed the younger adults on the Vocabulary subtest of the Shipley Institute of Living Scale (Shipley, 1986; *t*(62) = 3.47, *p* < .005, *d* = 0.881). Older adults were additionally screened for cognitive impairment using the Montreal Cognitive Assessment (MoCA; Nasreddine et al., 2005) and all participants performed within the normal range (*M* = 28.03, *SD* = 1.03). A summary of demographic information can be found in Table 1. Participants in all experiments provided written informed consent prior to beginning the experiment using methods approved by the Cambridge Psychology Research Ethics Committee, and received monetary compensation at a rate of £7.50 per hour for participation.[Table-anchor tbl1]

#### Materials

**Figure 1 fig1:**
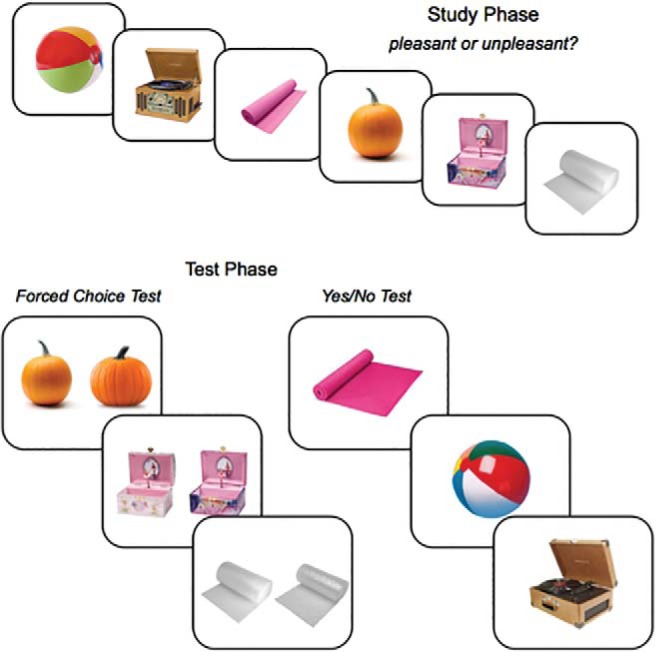
Experiment paradigm. Study phase schematic depicting examples of experimental stimuli (top) and test phase schematic depicting examples of Forced Choice and Yes/No test trials (bottom). The Yes/No test display depicts examples of ‘new’ trials containing similar foil objects.

A total of 800 color images of everyday objects were used as stimuli. This set consisted of 400 unique pairs of everyday object exemplars, collected from a combination of online sources, including Google Image Search (Mountain View, CA) and the stimulus sets available from the Konkle Lab (http://konklab.fas.harvard.edu). Each exemplar pair shared the same basic-level name (e.g., umbrella) and possessed a high degree of feature overlap (e.g., shape, color, pattern) such that targets and foils could not be discriminated without a detailed representation of each object (see Figure 1). To minimize any effects of pairwise variability in target-foil similarity on performance, an independent sample of participants rated exemplar pairs on perceptual similarity. We then created stimulus lists with equivalent levels of target-foil similarity on average, and counterbalanced the assignment of stimulus lists to test format and study block across participants.[Fig-anchor fig1]

#### Procedure

Each session began with a practice block in which participants completed an abbreviated version of the task that provided feedback on performance accuracy. This ensured that all participants understood the nature of the memory test, including the importance of memory for specific perceptual details of each stimulus for successful test performance. Each participant then completed two study-test blocks, with the procedure identical for each block of the experiment. A 5-min break divided the first and second block during which participants performed the Vocabulary subtest of the Shipley Institute of Living Scale. Older adults additionally completed the MoCA at the end of the testing session.

During each study phase, participants were presented with 200 pictures of everyday objects for 3000 ms each and asked to judge the pleasantness of each object. Participants were instructed to make this judgment based on the physical attributes of the stimulus (e.g., color, shape, pattern, and texture) to direct attention toward the perceptual features of each object, and to equate as much as possible the way in which stimuli were processed during the study phase across participants. After a 60-second filled interval during which participants counted backward by sevens from a random 3-digit number, participants completed a recognition memory test.

One half of the test comprised a Forced Choice format, wherein a target and its corresponding foil were presented simultaneously, one on the left of the screen and one on the right. The other half of the test was in a Yes/No format, wherein a single exemplar was presented in the center of the screen, which could be either a target or a foil. To equate the length of the Forced Choice and Yes/No tests, in the Yes/No test, either the target *or* its corresponding foil was presented. Accordingly, half of the stimuli were tested with the studied item as the test cue, and the other half tested with the corresponding foil as the cue, with this assignment counterbalanced across participants. Prior to the beginning of each test format, participants were reminded of the instructions and response options for that test. The order of the test formats was consistent across both blocks and counterbalanced across participants.

During each test phase, participants followed a modified Remember/Know procedure (Migo et al., 2009), indicating their recognition decision and the nature of their memory for the object by selecting from four response options. In the Forced Choice test, participants were instructed to select a ‘remember left’ or ‘remember right’ response if they could recall specific details of the exemplar they judged to have been previously studied, and a ‘familiar left’ or ‘familiar right’ response if their decision was based on greater familiarity of one exemplar over the other. In the Yes/No test, participants were instructed to select a ‘remember’ response if they recalled specific details of a studied exemplar and used these details to either accept a target (‘remember old’) or to reject a foil (‘remember new’). Participants were instructed to select a ‘familiar’ response if they were unable to recall specific details of a studied exemplar, and instead based their decision on the presence (‘familiar old’) or absence (‘unfamiliar new’) of familiarity for the presented object.

### Results

**Figure 2 fig2:**
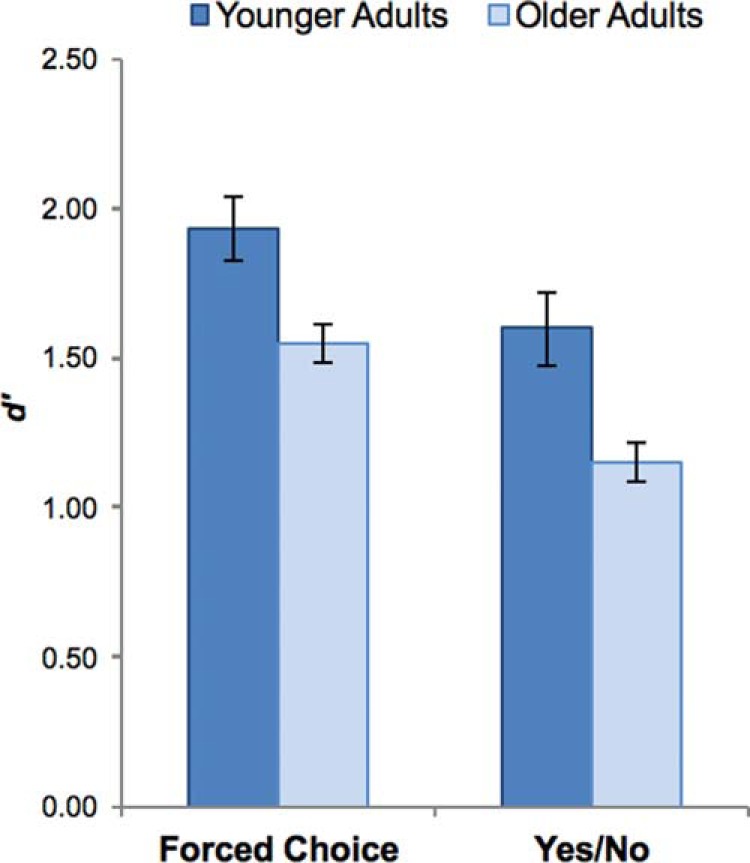
Recognition memory performance in the Forced Choice and Yes/No test formats in Experiment 1, collapsed across ‘remember’ and ‘familiar’ judgments. Both older and younger adults exhibited superior memory performance the Forced Choice test relative to the Yes/No test, although age differences in performance were observed across test formats. Error bars represent standard error.

We first compared recognition memory performance across age groups in the Forced Choice and Yes/No test formats, collapsing across remember and familiar responses (see Figure 2; raw proportions are shown in Table 2). We did this by computing *d*′ scores from the proportion of correct responses in the Forced Choice test and the proportion of hits and false alarms in the Yes/No test (Macmillan & Creelman, 1991), and submitting these scores to a 2 × 2 mixed ANOVA with Test Format (FC, YN) as a within-subjects factor and Age (young, old) as a between-subjects factor. The ANOVA revealed that both older and younger adults performed significantly better on the Forced Choice test relative to the Yes/No test, *F*(1, 62) = 45.61, *p* < .001, η_p_^2^ = 0.424, and that older adults performed significantly worse than younger adults across both test formats, *F*(1, 62) = 11.84, *p* < .005, η_p_^2^ = 0.160, with the size of this deficit equivalent across test formats (*F* < 1). [Fig-anchor fig2][Table-anchor tbl2]

We next sought to test the prediction that successful performance in the Yes/No test is driven primarily by the use of a recall-to-reject strategy, and that this strategy is impaired with age. To this end, we computed a ‘recall-to-reject’ measure calculated as the proportion of ‘remember new’ responses minus the proportion of ‘remember’ misses, and a ‘recall-to-accept’ measure calculated as the proportion of ‘remember old’ responses minus the proportion of ‘remember’ false alarms (Migo et al., 2009). A Response Type (reject, accept) × Age ANOVA revealed a main effect of Response Type, *F*(1, 62) = 6.51, *p* < .05, η_p_^2^ = 0.095, indicating a greater proportion of accurate responses were associated with the use of the ‘recall-to-reject’ strategy relative to a ‘recall-to-accept’ strategy. This did not interact with Age (*F* < 1), indicating that older adults’ performance also benefited from the use of a recall-to-reject strategy. However, a main effect of Age, *F*(1, 62) = 15.32, *p* < .001, η_p_^2^ = 0.198, indicated that older adults used both strategies less successfully than younger adults (reject: *t*(62) = 3.40, *p* < .001, *d* = 0.86; accept: *t*(62) = 3.35, *p* < .001, *d* = 0.85). Age-related deficits in the ability to use these strategies were driven by both an increase in ‘remember’ false alarms, *t*(62) = 3.21, *p* < .005, *d* = 0.82, and a reduction in the proportion of ‘remember’ correct rejections, *t*(62) = 3.23, *p* < .005, *d* = 0.82.

Next, we tested the prediction that the Forced Choice test format reduces demands on recollection-based retrieval strategies relative to the Yes/No test, enabling performance to be more successfully supported by familiarity-based judgments. To this end, we first conducted a 2 × 2 × 2 mixed ANOVA with Test Format (YN, FC) and Response Type (Remember, Familiar) as within-subjects factors and Age as a between-subjects factor on the proportion of correct responses to old items (i.e., hits in the Yes/No test, correct responses in the Forced Choice test). This revealed a main effect of Response Type, *F*(1, 62) = 295.58, *p* < .001, η_p_^2^ = 0.827, with more responses associated with ‘remember’ than ‘familiar’ responses across groups, which was qualified by a Test × Response Type interaction, *F*(1, 62) = 6.89, *p* < .05, η_p_^2^ = 0.100, that did not vary with age (*F* < 1). This reflected a greater contribution of recollection to correct ‘old’ responses in the Yes/No test than the Forced Choice test, *t*(63) = 2.38, *p* < .05, *d* = 0.57, but a greater contribution of familiarity to correct responses in the Forced Choice test than the Yes/No test, *t*(63) = 2.71, *p* < .01, *d* = 0.68. The main effect of Age and the Age × Response Type interaction were not significant (*F*s < 1), indicating older adults made similar proportion of correct remember and familiar responses to old items as did younger adults.

Next we conducted the same ANOVA on the proportion of incorrect old responses to similar foils (i.e., false alarms in the Yes/No test, incorrect responses in the Forced Choice test). This revealed a main effect of Test Format, such that more false alarms were made in the YN test than the FC test, *F*(1, 62) = 305.18, *p* < .001, η_p_^2^ = 0.831, and Response Type, such that false recognition was more often associated with ‘remember’ than ‘familiar’ responses, *F*(1, 62) = 4.93, *p* < .05, η_p_^2^ = 0.074, overall. This was qualified by a Test × Response Type interaction, *F*(1, 62) = 25.42, *p* < .001, η_p_^2^ = 0.291, reflecting a larger increase in the tendency to make ‘remember’ false alarms, *t*(63) = 14.49, *p* < .001, *d* = 3.65, than familiarity-based false alarms, *t*(63) = 9.05, *p* < .001, *d* = 2.28, in the Yes/No test relative to the Forced Choice test. There was also a main effect of Age, *F*(1, 62) = 13.78, *p* < .001, η_p_^2^ = 0.182, indicating older adults made more false alarms overall, although the interaction with Response Type did not reach significance (*p* = .11). Nonetheless, independent *t* tests indicated that older adults made more remember-based false alarms across test formats (YN: *t*(62) = 3.21, *p* < .005, *d* = 0.82; FC: *t*(62) = 2.59, *p* < .05, *d* = 0.66) but did not differ with respect to the number of familiarity based false alarms in either test format (YN: *t*(62) = 1.60, *p* = .11, *d* = 0.41; FC: *t* < 1).

### Discussion

The results of Experiment 1 revealed high rates of lure false recognition in the Yes/No test across both groups, which were reduced substantially by the simultaneous presentation of targets and foils in the Forced Choice test format. This observation is consistent with previous findings (Guerin et al., 2012; Migo et al., 2009), and with proposals that false recognition in the Yes/No test is often driven by a failure to retrieve stored details. This idea was further supported by the modified RK judgments, which indicated that accurate responding in the Yes/No test relies primarily on the successful application of a recall-to-reject strategy, whereas familiarity can more successfully support Forced Choice decisions, again consistent with previous work (Migo et al., 2009).

Interestingly, we found that incorrect responses were more often associated with ‘remember’ judgments across age groups. This pattern suggests that memory errors were typically associated with illusory recollection, wherein the subjective experience of remembering accompanies inaccurate responses. This tendency is not uncommon in recognition memory tests with highly similar foils, and has been observed previously among healthy younger adults (Kim & Yassa, 2013). This may arise from an increased likelihood for participants to think a recollected detail is diagnostic of the target, when in fact is it shared by targets and foils (Migo et al., 2009). Alternatively, participants may have tended to misattribute a spontaneously activated prototypical feature of an item as having been studied, or erroneously recombined studied features from one object and another (Doss, Bluestone, & Gallo, 2016). Notably, the incidence of illusory recollection was considerably greater in the Yes/No test than the Forced Choice test, consistent with increased demands on postretrieval monitoring and evaluation of retrieved details in this test format.

As predicted, older adults were significantly impaired in the ability to execute a recall-to-reject strategy, as well as a recall-to-accept strategy. This was driven by an increase in lure false alarms, coupled with an intact hit rate to studied items, replicating previous work (Koutstaal, 2003; Stark et al., 2015; Toner et al., 2009; Yassa et al., 2011). These findings are consistent with existing proposals that aging is associated with declines in the ability to use a recall-to-reject strategy in other domains, such as source exclusion tasks (Gallo et al., 2006) and associative recognition tests (Cohn et al., 2008). Interestingly, older adults’ false alarms were primarily associated with incorrect ‘remember’ responses, consistent with previous observations that increased false recognition with age is more often accompanied by high confidence, illusory recollection than increased reliance on familiarity (Dodson, Bawa, & Slotnick, 2007; see McCabe, Roediger, McDaniel, & Balota, 2009 for review). This tendency is thought to reflect impairments in postretrieval monitoring and evaluation processes with age (Dodson, Bawa, & Krueger, 2007; Gallo et al., 2006; Wong, Cramer, & Gallo, 2012), and may arise, at least in part, as a result of reductions in the availability of object information that can be used to detect a mismatch between targets and lures.

Consistent with this possibility, although older adults displayed similar benefits to performance from the reinstatement of target details in the Forced Choice test as younger adults, age differences in this test format were still observed. This deficit indicates that impairments in strategic retrieval processes alone cannot account for older adults’ performance, and suggests an age-related decline in availability of stimulus details that can disambiguate targets and foils, as suggested by previous work (Burke et al., 2010, 2011; Ryan et al., 2012; Yeung et al., 2013). These results raise an important possibility. If object details that can successful disqualify lures are less available to older adults, this factor alone could be driving age-related impairments in the use of a recall-to-reject strategy in the Yes/No test by reducing older adults’ ability to detect a mismatch between targets and foils. Alternatively, it may be the case that even if the availability of object details were equated across groups, older adults would continue to exhibit deficits in Yes/No performance due to impairments in the ability to strategically retrieve and evaluate these details. We aimed to tease apart these possibilities in Experiment 2.

## Experiment 2

Existing empirical and modeling work suggests that the ability to discriminate between perceptually similar object exemplars can be impacted by the presence of interference from objects sharing common lower-level features (Cowell et al., 2006). For example, studies in healthy younger adults have found that Forced Choice object recognition is reduced after viewing visual interference containing objects, but not analogous interference comprised of scenes (Watson & Lee, 2013; O’Neil, Watson, Dhillon, Lobaugh, & Lee, 2015). In contrast, existing work in healthy older and younger adults has found that varying the number of objects between study and test in a Yes/No recognition memory test did not affect performance in either age group (Stark et al., 2015). These findings suggest that Forced Choice performance may be more directly impacted by the presence of interference from objects sharing lower level features than Yes/No performance. Furthermore, older adults may be more susceptible to feature-level interference than younger adults (Burke et al., 2012; Newsome et al., 2012; Ryan et al., 2012; Yeung et al., 2013), raising the possibility that this factor exacerbated age differences in performance in Experiment 1.

To our knowledge, no work to date has compared the effects of interference across test formats, or how this might impact the presence of age differences in each case. In Experiment 2, we explored this question by assessing older and younger adults’ performance in each test format while varying the number of objects in the study list across study-test cycles. We varied the length of each study list differently according to age group, such that the longer study list for older adults was the same length as the shorter list in younger adults. This enabled us to compare performance across groups when older adults faced an equivalent amount of interference relative to younger adults, as well as when they faced reduced interference relative to younger adults.

If Forced Choice performance is disproportionately affected by interference from viewed objects, and older adults are more vulnerable to interference than younger adults, reducing the number of studied objects may ameliorate age differences in this test format. If so, this will enable us to assess whether age differences in Yes/No performance continue. Persistent deficits in Yes/No performance would suggest that age differences cannot be explained solely by reductions in the availability of object details, implicating additional contributions of impaired strategic retrieval processes. In contrast, if performance improves similarly across test formats, this would suggest a single factor, namely reductions in the availability of object details, drives increased false recognition across test formats.

### Method

#### Participants

A new group of 34 younger adults aged 18 – 28 years (*M* = 21.74, *SD* = 2.39) and 48 older adults aged 60–80 years (*M* = 70.29, *SD* = 5.86) participated in this experiment. All participants were native English speakers. The younger adults were students from the University of Cambridge and the older adults were healthy, community-dwelling volunteers. Older and younger adults did not differ with respect to years of formal education (*t* < 1) and older adults scored significantly higher on the Vocabulary subtest of the Shipley Institute of Living Scale, *t*(77) = 5.77, *p* < .001, *d* = 1.315. Three older adults were excluded from the analyses because they performed below the normal range (<26) on the MoCA, leaving 45 healthy older adults who performed well within the normal range (*M* = 28.11, *SD* = 1.19). A summary of demographic information can be found in Table 1.

#### Materials

A total of 960 color images of everyday objects were used as stimuli. This set consisted of the 800 images used in Experiment 1, plus an additional 160 images obtained from similar sources to produce 480 unique pairs of object exemplars. As in Experiment 1, each exemplar in a pair served equally often as the studied target and unstudied foil. These object pairs were divided into eight 60-item lists, with the allocation of each list to the short and long study lists and to the Forced Choice and Yes/No test format counterbalanced across participants. The length of the short and long lists differed for each age group, such that younger adults studied 180 items in their short block and 300 items in their long block, whereas older adults studied 60 items in their short block and 180 items in their long block. This resulted in a reference block of the same length completed by both groups, coupled with a block that was shorter or longer than the reference block for older and younger adults, respectively. The length of each list was selected so as to create two lists that were maximally different in length, where the difference in length was equivalent across age groups (in this case the lists differed by 120 items), with the reference block length as close as possible to the list length used in Experiment 1.

#### Procedure

The procedure for this experiment was identical to that of Experiment 1, with the following exceptions. Participants completed two study-test blocks of unequal length, with length scaled for each age group, as described above. The order of the short and long blocks was counterbalanced across participants. A 10-min break was provided between blocks one and two during which participants were asked to rest quietly, to minimize the possibility of carry-over effects of interference from block one to block two. The test phase was again divided into a Forced Choice Test and a Yes/No Test, with test order consistent across blocks and counterbalanced across participants. Unlike Experiment 1, participants made simple Left/Right and Yes/No decisions in the Forced Choice and Yes/No formats, respectively. The modified Remember/Know judgments were removed from this test to simplify the response options.

### Results

**Figure 3 fig3:**
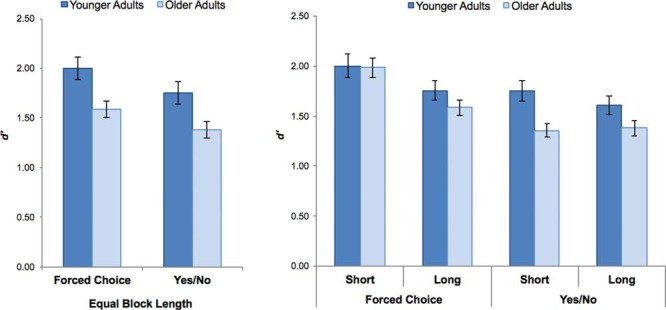
(Left) Recognition memory performance following study of an equal number of items (180 objects) across age groups in Experiment 2. Age differences in performance were observed across test formats. (Right) Recognition memory performance following short (YA: 180 objects; OA: 60 objects) and long (YA: 300 objects; OA: 180 objects) study-test blocks in each test format. Age differences are observed in the Yes/No test, but not the Forced Choice test. Error bars represent standard error.

As in Experiment 1, we computed *d*′ scores to compare older and younger adults’ recognition memory performance in the Forced Choice and Yes/No Tests at each block length (Macmillan & Creelman, 1991). Before exploring the effects of interference on performance, we first assessed performance when our two groups faced an equivalent amount of interference, namely for the reference block containing 180 stimuli, which is depicted in Figure 3 (left; raw scores in Table 3). To this end, we conducted a 2 × 2 mixed ANOVA with Test Format (FC, YN) as a within-subject factor and Age (young, old) as a between-subjects factor. The results replicated those observed in Experiment 1, with (a) both older and younger adults performing significantly better in the Forced Choice test relative to the Yes/No test, *F*(1, 77) = 15.15, *p* < .001, η_p_^2^ = 0.164, (b) older adults performing significantly worse than younger adults across both test formats, *F*(1, 77) = 10.13, *p* < .005, η_p_^2^ = 0.116, and (c) the size of this age effect being equivalent across test formats (*F* < 1). [Fig-anchor fig3][Table-anchor tbl3]

We next examined the effects of increasing interference, that is, increasing block length from 60 items to 180 items in older adults and from 180 to 300 items in younger adults (see Figure 3, right; raw scores in Table 3). To do so, we submitted participants’ *d*′ scores to a 2 × 2 × 2 mixed ANOVA with Block Length (short, long) and Test Format as within-subject factors and Age as a between-subjects factor. The ANOVA revealed significant main effects of Block Length, *F*(1, 77) = 18.80, *p* < .001, η_p_^2^ = 0.1960, Test Format, *F*(1, 77) = 42.77, *p* < .001, η_p_^2^ = 0.357, and a marginal effect of Age, *F*(1, 77) = 3.48, *p* = .066, η_p_^2^ = 0.043. These main effects were qualified by a significant Block Length × Test Format interaction, *F*(1, 77) = 8.49, *p* < .005, η_p_^2^ = 0.099, and Test Format × Age interaction, *F*(1, 77) = 5.38, *p* < .05, η_p_^2^ = 0.065. To investigate how the effects of Block Length and Age varied across Test Format, we conducted follow-up Block Length × Age ANOVAs separately for Forced Choice and Yes/No Test performance.

In the Forced Choice Test, we observed a significant main effect of Block Length, *F*(1, 77) = 26.57, *p* < .001, η_p_^2^ = 0.257, that did not differ with age, *F*(1, 77) = 1.59, *p* = .211, η_p_^2^ = 0.020. Critically, the effect of age on recognition memory performance was not significant (*F* < 1), and this was true for both short (*t* < 1) and long, *t*(77) = 1.37, *p* = .17, block lengths. In contrast, Yes/No performance did not decline significantly as block length increased (*F* < 1), and this was true across both age groups, with no evidence of an interaction, *F*(1, 77) = 1.67, *p* = .20, η_p_^2^ = 0.021. However, significant age differences in Yes/No performance persisted, *F*(1, 77) = 7.63, *p* < .01, η_p_^2^ = 0.090.

### Discussion

The results of Experiment 2 revealed dissociable effects of the list length manipulation across test formats. In both groups, performance in the Forced Choice test was modulated by the number of studied items, whereas performance in the Yes/No test remained stable across list lengths. This observation is consistent with previous work identifying effects of object interference on Forced Choice performance (O’Neil et al., 2015; Watson & Lee, 2013), but not on Yes/No performance (Stark et al., 2015), and lends support to the proposal that distinct mechanisms support memory performance across test formats. In particular, this observation suggests that Forced Choice performance is more directly related to the availability of object information, and therefore affected by the presence of interference from increased exposure to objects sharing common features. In contrast, Yes/No performance may be constrained by one’s ability to successfully execute recollection-based retrieval processes, which may place an upper boundary on performance. Similarly, previous work has suggested that familiarity-based memory performance may be more sensitive to the effects of interference than recollection-based memory (Sadeh, Ozubko, Winocur, & Moscovitch, 2016), consistent with the proposal that familiarity and recollection differentially contribute to performance in the Forced Choice and Yes/No test formats, respectively (Migo et al., 2009, 2014; Norman, 2010).

Notably, we found that reducing list length eliminated age differences in Forced Choice performance, whereas age difference in Yes/No performance persisted. The observation that reducing exposure to objects sharing overlapping features enhanced older adults’ Forced Choice performance may reflect an increased vulnerability to interference with age, as suggested by previous work (Burke et al., 2012; Newsome et al., 2012; Ryan et al., 2012; Yeung et al., 2013), which is consistent with an age-related decline in the availability of object information. In support of this possibility, as the number of studied objects increased, older adults exhibited a similar decline in performance as younger adults, but in the face of a considerably smaller amount of object interference. Accordingly, when we examined conditions that were analogous to those of Experiment 1, in which list length was matched across groups, older adults exhibited deficits across both test formats, replicating our prior results. Importantly, the observation of persistent deficits in Yes/No performance suggests that this single factor is unlikely to fully account for age differences in the Yes/No test. Instead, the current results point to an additional contribution of impaired strategic retrieval processes to age differences in Yes/No performance. This observation is consistent with existing evidence for disproportionate age differences in memory performance when demands on these strategies (i.e., recall-to-reject) are high (Cohn et al., 2008; Gallo et al., 2006; Luo & Craik, 2009).

An important caveat to these interpretations is that we did not examine the effects of different types of interference on memory performance across test formats. Accordingly, we cannot be certain that the effects of increasing list length on Forced Choice performance is related to an increase in interference from objects sharing lower-level features. Moreover, the list length manipulation not only altered the amount of object exposure, but also affected the memory load and duration of the study phase. Thus, we cannot rule out the possibility that other factors, such as increased attentional demands or fatigue associated with studying more items, contributed to this observation. However, the selective effect of the list length manipulation on performance in the Forced Choice test argues against these explanations, as such effects would be expected to impact both test formats in a similar fashion. Irrespective of the specific mechanism that led to the pattern of results observed here, the selective effect of the list length manipulation on Forced Choice performance lends support to the possibility that partially distinct factors determine performance across test formats. Future work should assess whether the current pattern also extends to different forms of interfering information, or is specific to objects that share common features.

## Neuropsychological Assessment

The results of Experiments 1 and 2 suggest that elevated rates of lure false recognition with age arise as a result of contributions of both reductions in the availability of object details with age, and impairments in the ability to carry out strategic retrieval processes. However, existing work suggests that these factors may not be affected to a similar degree across older adults (Davidson & Glisky, 2002; Glisky, Rubin, & Davidson, 2001; Migo et al., 2014; Toner et al., 2009). Thus, it may be the case that individual differences in the availability of object details and the ability to execute strategic retrieval processes will impact the susceptibility to false recognition across older adults. Furthermore, if the Forced Choice and Yes/No test formats are differentially sensitive to each of these factors, as suggested by prior work (Guerin et al., 2012; Migo et al., 2009, 2014; Norman, 2010), individual variability in these measures may differentially impact performance in each test format. To investigate these possibilities, we explored the relationship between individual differences in strategic retrieval processes and the availability of object details in relation to older adults’ performance across test formats.

To capture individual differences in strategic retrieval processes that are necessary to support a recall-to-reject strategy, we included measures of executive function and recall ability, which place common demands on cognitive control processes such as selection, inhibition, and maintenance of stored details. To assess the availability of object details, we used a perceptual task that involves discriminating between objects with overlapping features, thus placing similar demands on the type of object representation needed to support the task, but eliminating any mnemonic demands. We predicted that perceptual discrimination ability would be selectively related to performance in the Forced Choice test, based on the proposal that the Forced Choice test is more sensitive to the availability of object details relative to the Yes/No test. In contrast, we predicted that performance in the Yes/No test would be related to executive function and recall ability, which are critical components of executing a recall-to-reject strategy.

### Method

#### Participants

Forty-two older adults who participated in Experiments 1and 2 returned to the lab to complete a neuropsychological testing battery within 12 months of completing the initial experiment. These participants were randomly selected from the sample of older adults that completed Experiments 1 and 2, with the constraint that individuals were drawn from the full range of performance on the task. The older adults from each experiment did not differ with respect to mean age, years of education, or Shipley Vocabulary Scores (all *t* < 1). The groups did differ in their scores on the Montreal Cognitive Assessment, however both groups performed well within the normal range (Exp. 1: *M* = 28.25, *SD* = 1.12; Exp. 2: *M* = 27.23, *SD* = 1.69; *t*(40) = 2.29, *p* < .05, *d* = 0.724). The individuals from each experiment were also matched on object recognition memory performance across test formats (all *p* > .2) and were combined for all subsequent analyses. A summary of the demographic information can be found in Table 4.[Table-anchor tbl4]

#### Neuropsychological battery & procedure

All participants completed a battery of neuropsychological tests assessing memory, executive function, and perception. These included the Logical Memory and Paired Associates subtests from the Wechsler Memory Scale (3rd Edition [WMS-III]; Wechsler, 1997a), the Rey-Osterrieth Complex Figure Test (Osterrieth, 1944), the Verbal Fluency and Trails A & B subtests from D-KEFS (Delis, Kaplan, & Kramer, 2001), and the Digit Span subtest from the Wechsler Adult Intelligence Scale (3rd Edition [WAIS-III]; Wechsler, 1997b). Participants additionally completed a complex visual discrimination task using stimuli developed by Barense and colleagues (2012; see also Newsome et al., 2012 and Ryan et al., 2012). In this task, participants are simultaneously presented with two novel objects and decide if they match or do not match. Critically, when these objects share multiple overlapping features (e.g., high ambiguity trials), convergent evidence from patients with PRC lesions (Barense et al., 2012) and neuroimaging studies in older (Ryan et al., 2012) and younger (Barense et al., 2012) adults indicates that successful performance relies on object-level representations supported by the PRC to resolve feature level ambiguity between exemplars.

Scores on each of these subtests were normalized and the *z* scores averaged to create three different composite scores for each individual: a Representational Quality score, a Recall Performance score, and an Executive Function score. The Representational Quality score consisted of performance on the high ambiguity condition of the visual discrimination task. The Recall Performance score comprised immediate and delayed recall scores from the Logical Memory, Paired Associates, and Rey Complex Figure tests. The Executive Function score comprised Verbal Fluency, Trails B, and Digit Span scores. The group was median split on each composite score to divide participants into high and low scoring groups in each of the three factors. One-way between-participants analyses of variance confirmed that the high and low scoring groups differed significantly on their respective composite scores (Representational Quality Groups: *F*(1, 41) = 74.69 *p* < .001; Recall Performance Groups: *F*(1, 41) = 76.13, *p* < .001; Executive Function Groups: *F*(1, 41) = 49.34 *p* < .001). A full summary of the demographic information and composite scores for each group can be found in Table 5.[Table-anchor tbl5]

### Results

**Figure 4 fig4:**
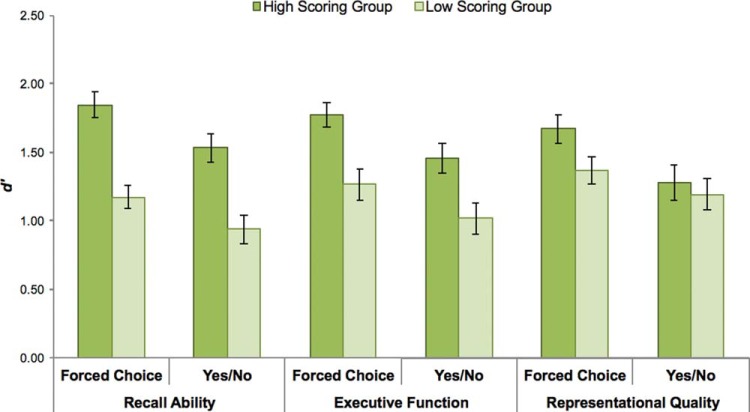
Recognition memory performance among older adults divided into low and high scoring groups based on neuropsychological test performance. Older adults with higher scores in Executive Function and Recall Ability performed significantly better across test formats than older adults with lower scores in these measures. Older adults with higher scores in Representational Quality performed significantly better than older adults with lower scores in this measure in the Forced Choice test, but these groups did not differ significantly in Yes/No test performance. Error bars represent standard error.

We first sought to assess the degree to which the high or low scoring group in each of the three cognitive factors of interest differed in performance across test formats (see Figure 4). To this end, we submitted participants’ *d*′ scores from the Forced Choice and Yes/No test formats to three mixed ANOVAs with Test Format as a within-subjects factor and Group (high vs. low scoring) as a between-subjects factor. For those participants who completed Experiment 2, we used performance on the 180 item block for their *d*′ scores to ensure that performance measures were based on comparable experimental conditions across participants.[Fig-anchor fig4]

We found that those older adults scoring higher in Recall Ability performed significantly better than those individuals in the low scoring group, *F*(1, 40) = 23.66, *p* < .001, η_p_^2^ = 0.372, across both test formats (*F* < 1). This effect remained significant when Executive Function score was taken into account, *F*(1, 40) = 5.71, *p* < .05, η_p_^2^ = 0.144. Similarly, we observed a significant difference in memory performance between the High and Low Executive Function groups, *F*(1, 40) = 11.07, *p* < .005, η_p_^2^ = 0.217, across test formats (*F* < 1), and this effect remained when Recall Ability was included as a covariate, *F*(1, 40) = 4.35, *p* < .05, η_p_^2^ = 0.100. In contrast, those older adults who scored high and low in Representational Quality did not differ with respect to overall memory performance (*F* < 1). Instead, we observed a significant Test Format × Group interaction, *F*(1, 40) = 4.42, *p* < .05, η_p_^2^ = 0.100, which reflected a selective impact of Representational Quality on Forced Choice performance, *t*(40) = 2.07, *p* < .05, *d* = 0.638, which was not observed in Yes/No performance (*t* < 1). Critically, this interaction remained significant when Recall Score and Executive Function were included as covariates in the ANOVA, *F*(1, 40) = 4.12, *p* < .05, η_p_^2^ = 0.098, indicating an impact of Representational Quality on Forced Choice performance even after the effects of recall and executive function have been accounted for.

We next sought to assess whether the benefit to performance gained by the simultaneous presentation of targets and foils in the Forced Choice test was constrained by individual differences in representational quality. To explore this possibility, we computed a difference score quantifying the memory enhancement associated with the Forced Choice test compared with the Yes/No test (see also Westerberg et al., 2013). We then assessed whether the size of this mnemonic benefit varied according to group membership for the three neuropsychological factors. Consistent with predictions, this analysis revealed that those participants in the high Representational Quality group benefited significantly more from the presence of retrieval support than did the low Representational Quality group, *t*(40) = 2.10, *p* < .05, *d* = 0.664, whereas there was no significant difference in this benefit between high and low scoring participants in the Recall Performance or Executive Function groups (*t*s < 1).

### Discussion

Consistent with our first prediction, older adults with higher scores in representational quality exhibited superior Forced Choice performance, and exhibited larger benefits of the simultaneous presentation of targets and foils in the Forced Choice test, relative to those individuals scoring low in this measure. Critically, this relationship was observed even after accounting for individual differences in recall ability and executive function, consistent with the proposal that the Forced Choice test format enables a direct assessment of the availability of object details. The observation that the size of the Forced Choice benefit was selectively constrained by this factor is also consistent with the proposal that this test format minimizes demands on strategic retrieval processes, resulting in the availability of object details having a larger impact on performance. In contrast, we found that representational quality did not have a direct effect on performance in the Yes/No test, suggesting that the availability of object details is not sufficient to support a recall-to-reject strategy, likely because of the additional demands this test places on the ability to strategically retrieve, maintain, and evaluate stored details.

Consistent with this possibility, and our second prediction, older adults who scored higher on measures of both recall ability and executive function performed significantly better in the Yes/No test. This observation replicates previous work identifying a relationship between Yes/No recognition performance with similar foils and a measure of recall ability in older adults (Holden et al., 2013; Migo et al., 2014; Toner et al., 2009), and extends this work by identifying a similar relationship for executive function. These relationships are consistent with the demands this test format places on using a recall-to-reject strategy, which involves cognitive control processes such as selection, inhibition, and postretrieval monitoring that are captured by measures of recall and executive function. Interestingly, those older adults scoring higher on these factors also performed significantly better on the Forced Choice test than those with lower scores. Although we did not predict this relationship, it may reflect the general benefit of memory control processes, which are common to recall and executive function, on memory performance.

## General Discussion

The current investigation explored the degree to which impairments in the ability to strategically retrieve object details, relative to declines in the availability of these details, contribute to elevated rates of lure false recognition with age. To this end, we assessed older and younger adults’ recognition memory performance in both Yes/No and Forced Choice test formats, on the basis that the simultaneous presentation of targets and foils in the Forced Choice test minimizes demands on strategic retrieval processes, enabling a more direct measure of the availability of object details (Guerin et al., 2012; Migo et al., 2009; Norman, 2010). The results of Experiment 1 revealed that age-related increases in false recognition were evident across test formats, implicating reductions in the availability of object details to increased false recognition among older adults. Experiment 2 assessed whether this factor alone could be driving false recognition across test formats, but found that age differences in Yes/No performance persisted even when performance in the Forced Choice test was matched across groups. Together, these results indicate that both impairments in strategic retrieval processes and reductions in the availability of object details contribute to elevated rates of false recognition with age.

The present results complement existing research exploring gist-based false recognition in younger adults by identifying evidence for a substantial contribution of retrieval failure to false recognition of similar lures (Guerin et al., 2012; Migo et al., 2009). Specifically, we found that the incidence of false recognition was considerably reduced in the Forced Choice test format relative to the Yes/No format, indicating that the object details necessary to discriminate between targets and lures are often available in memory, even when they are not retrieved successfully. Importantly, the present observation that older adults benefited to the same degree as younger adults from the simultaneous presentation of targets and foils in the Forced Choice test lends further support to the proposal that this test format improves the accessibility of stored details, and does so across the life span. This enhancement likely arises because performance in the Forced Choice test can more successfully be supported by assessing the relative familiarity between exemplars, whereas Yes/No performance relies critically on a recall-to-reject strategy (Migo et al., 2009; Norman, 2010), a possibility that is supported by the results of the modified Remember-Know judgments included in Experiment 1.

Critically, the present results also extend previous work by providing evidence that increases in lure false recognition among older adults cannot be explained solely by retrieval failure, and are in part the result of declines in the availability of object details that can successfully disambiguate targets and foils with overlapping features. This observation is consistent with recent evidence for age-related decline in the ability to discriminate between objects that share overlapping features, even when demands on explicit memory encoding and retrieval are minimized (Burke et al., 2010; Lee et al., 2014; Ryan et al., 2012; Yeung et al., 2013). Together with the present results, this evidence suggests that aging is associated with decline in the quality of online stimulus representations, such that these representations are less able to disambiguate targets and foils that share overlapping features. More specifically, these data are consistent with recent proposals that aging is associated with a reduction in the availability of unique object-level representations, leading to increased reliance on representations of simple features and feature conjunctions that comprise these objects, which remain unaffected (Burke et al., 2010, 2011; 2012; Ryan et al., 2012).

This proposal is based on the representational-hierarchical framework, which states that increasingly complex stimulus representations are supported along the posterior-anterior axis of the ventral visual stream, from simple features and feature conjunctions, to the level of a unique object (Bussey & Saksida, 2007; Cowell et al., 2006). According to this view, object-level representations are critical for resolving feature ambiguity between objects sharing overlapping features and are supported by the perirhinal cortex (PRC). When these representations become compromised as a result of damage or dysfunction of PRC, as may occur with increased age (see Burke et al., 2012 and Leal & Yassa, 2015 for reviews), individuals must rely on simple, feature-level representations that are less able to disambiguate targets and foils with overlapping features, resulting in increased false recognition of objects that share common lower level features. Critically, this model makes three specific predictions that are supported by the present data: (a) discrimination between targets and foils with overlapping features should be impaired, even when demands on controlled retrieval processes are minimized, (b) impaired performance arises due to increased vulnerability to feature-level interference, and can be ameliorated by reducing feature-level interference, and (c) common representations support perceptual and mnemonic discriminations of object exemplars sharing overlapping features.

In support of these predictions, we identified age-related deficits in Forced Choice performance, as well as evidence that reducing the number of objects viewed by participants can enhance performance in this test format, perhaps by reducing interference from features shared across objects. Furthermore, we identified a relationship between performance in a perceptual discrimination task and Forced Choice performance. This finding provides novel evidence for an association between complex perception and memory ability among older adults, lending support to the proposal that common representations support memory and perception. Finally, although we did not obtain direct measures of PRC structure or function in the present experiment, the current results are nevertheless consistent with the possibility that performance in the perceptual discrimination task and Forced Choice test necessitate object-level representations supported by PRC. In particular, performance in the perceptual discrimination task used here has been linked to PRC recruitment among older adults using fMRI (Ryan et al., 2012), and is impaired in patients with compromised PRC integrity (Barense et al., 2012; Newsome et al., 2012). These findings raise the possibility that our measure of representational quality is sensitive to PRC function.

Consistent with this possibility, the relationship between performance in the perceptual discrimination task and Forced Choice performance in the present study bears a striking resemblance to that observed previously among patients with MCI and AD using a direct measure of PRC volume. In particular, PRC volume was selectively related to performance in the Forced Choice test format, as well as the benefit to performance individuals gained in the Forced Choice test relative to the Yes/No test, but not to performance in the Yes/No test format (Westerberg et al., 2013). The correspondence between these findings and the current results is consistent with a contribution of object-level representations supported by PRC to performance in both the perceptual discrimination task and Forced Choice test used here, and lends further support to the possibility that variability in PRC function may contribute to individual differences in Forced Choice performance in the present study.

Notably, although the availability of object details that can disambiguate targets and foils is a critical prerequisite for accurate memory performance across test formats, we did not identify a relationship between this factor and Yes/No performance. Instead, we found that performance in this task was related to tests measuring recall and executive function. Importantly, the tasks used in the current battery are thought to be sensitive to hippocampal and prefrontal function, respectively, and may reflect variability in the integrity of these regions in the current sample. This possibility is consistent with the role of the prefrontal cortex in selection, inhibition, maintenance and evaluation of stored details (Badre & Wagner, 2007), and the hippocampus in supporting reinstatement of previous episodes (i.e., pattern completion) and mismatch detection (i.e., pattern separation; Norman & O’Reilly, 2003), which are jointly thought to support a recall-to-reject strategy (Bowman & Dennis, 2016; Gallo, 2004). Importantly, the observation that representational quality was directly related to Forced Choice performance, but not Yes/No performance, suggests that the availability of object-level representations supported by PRC are necessary, but not sufficient, to support performance in the Yes/No test, making it difficult to detect measurable effects of representational quality on Yes/No performance.

Consistent with this possibility, previous work using fMRI has found that although PRC is recruited during a Yes/No recognition task with similar lures, only hippocampal activity is related to accurate performance (Reagh & Yassa, 2014). In contrast, PRC activity has been directly related to discrimination performance in a Forced Choice test with similar lures (O’Neil et al., 2015). Similarly, among patients with MTL damage, hippocampal integrity has been related to performance in Yes/No performance, whereas PRC integrity has been associated with Forced Choice performance (Holdstock et al., 2002; Westerberg et al., 2013). Collectively, this empirical evidence indicates that partially dissociable neural mechanisms can support performance across test formats when targets and foils are perceptually similar, consistent with modeling work (Norman, 2010). These direct neural measures complement the present behavioral findings in indicating that the Forced Choice test provides a more direct measure of the quality of object representations and underlying PRC function, relative to the Yes/No test. In doing so, these data further validate the use of the Forced Choice and Yes/No test formats to tease apart contributions of the availability of object details, relative to the ability to retrieve and evaluate these details, to elevated rates of false recognition among older adults.

Although we believe these two factors represent the most parsimonious explanation for the present results, we cannot rule out the potential contribution of age-related changes during encoding. Indeed, an important challenge associated with explicit memory tests is the difficulty of separating contributions of processes operating during encoding to the resulting quality of stimulus representations. For example, it is possible that older adults are more likely than younger adults to preferentially focus on semantic as compared with perceptual object information during encoding, thus reducing the availability of perceptual details at test and increasing reliance on semantic gist (Brainerd & Reyna, 2002; Koutstaal et al., 1997, 2003). Although we tried to minimize this possibility by using a perceptually oriented encoding task to encourage comparable processing of images by older and younger adults, it is difficult to completely rule out when concrete, meaningful objects are used as stimuli. Nevertheless, existing evidence argues against the idea that age differences during encoding, and in particular a tendency to predominantly focus on semantic information at the expense of perceptual detail, can account for the pattern of results presented here, as elaborated below.

First, existing work has identified age-related deficits in the ability to discriminate between items with overlapping features in the context of recognition memory tests, as well as perceptual discrimination tasks, using both abstract objects (Ryan et al., 2012; Pidgeon & Morcom, 2014) and unfamiliar faces (Bartlett, Leslie, Tubbs, & Fulton, 1989; Crook & Larrabee, 1992; Lee et al., 2014; Megreya & Bindemann, 2015). Such findings suggest that age differences in target-foil discrimination are not specific to stimuli that possess semantic meaning, nor task conditions with explicit ‘encoding’ and ‘retrieval’ phases, but rather any task that involves disambiguating objects with overlapping features. Furthermore, age-related deficits in perceptual discrimination and recognition memory of complex objects that share common features have been observed in aged rats and nonhuman primates (Burke et al., 2010, 2011; see Burke et al., 2012 for review), indicating that these deficits can emerge even when semantic meaning and explicit encoding strategies are unlikely to contribute to performance. Collectively, these findings are consistent with declines in the availability of object-level representations with age, resulting in impaired discrimination of items sharing overlapping features, thus lending further support to this interpretation of the present results.

In summary, the results of the current investigation provide evidence for the contribution of two factors to elevated rates of lure false recognition with age: declines in the availability of object details, and impairments in the strategic retrieval and evaluation of these details. Importantly, they also identify two ways in which false recognition can be reduced, including minimizing demands on strategic retrieval processes by increasing environmental support at test, and reducing interference from visual inputs that share common features with to-be-remembered information. The observation that age-related increases in false recognition can be minimized, or even eliminated, in this way may have implications for reducing everyday memory errors among older adults, as well as applications to legal domains such as eyewitness testimony (e.g., sequential vs. simultaneous line-ups; Mickes, Flowe, & Wixted, 2012; Wixted & Mickes, 2014). Finally, the current findings indicate that although false memory errors can increase dramatically and robustly with age, the susceptibility to lure false recognition varies substantially across older adults. Such observations highlight the importance of adopting an individual differences approach to investigations of memory decline in the elderly population.

## Figures and Tables

**Table 1 tbl1:** Demographic Information for Participants From Experiments 1 and 2

	Experiment 1		Experiment 2	
	Younger adults	Older adults	Younger adults	Older adults
*N*	32 (18F)	32 (16F)	34 (17F)	45 (21F)
Age	22.66 (3.0)	70.47 (4.6)	21.74 (2.4)	69.76 (5.6)
Education	16.72 (2.1)	17.81 (6.0)	16.08 (1.9)	16.47 (3.2)
Shipley	35.50 (2.3)	37.41 (2.1)	34.09 (3.2)	37.51 (2.1)
MoCA	—	28.03 (1.0)	—	28.11 (1.2)
*Note.* Standard deviations are indicated in parentheses next to mean values. MoCA = Montreal Cognitive Assessment; Shipley = Vocabulary subtest of the Shipley Institute of Living Scale.				

**Table 2 tbl2:** Proportion of ‘Remember’ and ‘Familiar’ Judgments by Response Accuracy in Experiment 1

Response Type	Younger adults	Older adults
Forced Choice Test		
Correct ‘Remember’	.68 (.17)	.67 (.13)
Correct ‘Familiar’	.20 (.13)	.18 (.12)
Incorrect ‘Remember’	.04 (.05)	.08 (.06)
Incorrect ‘Familiar’	.07 (.06)	.07 (.04)
Yes/No Test		
‘Remember’ Hits	.73 (.14)	.71 (.17)
‘Familiar’ Hits	.15 (.13)	.17 (.14)
‘Remember’ False Alarms	.22 (.13)	.33 (.14)
‘Familiar’ False Alarms	.17 (.11)	.21 (.12)
‘Remember’ Correct Rejections	.44 (.20)	.30 (.15)
‘Unfamiliar’ Correct Rejections	.17 (.14)	.15 (.12)
‘Remember’ Misses	.06 (.05)	.05 (.06)
‘Unfamiliar’ Misses	.07 (.02)	.06 (.01)
*Note.* Standard deviations are indicated in parentheses next to mean values.		

**Table 3 tbl3:** Raw Average Scores by Test Format and Block Length in Experiment 2

Condition	Younger adults	Older adults
Forced Choice Test		
Short Block		
Proportion Correct	.90 (.08)	.88 (.08)
Long Block		
Proportion Correct	.87 (.08)	.84 (.08)
Yes/No Test		
Short Block		
Hits	.86 (.09)	.87 (.09)
False Alarms	.33 (.12)	.42 (.14)
Long Block		
Hits	.84 (.10)	.87 (.08)
False Alarms	.33 (.09)	.45 (.12)
*Note.* Standard deviations are indicated in parentheses next to mean values.		

**Table 4 tbl4:** Demographics and Memory Performance of Older Adults Who Completed Neuropsychological Testing

	Experiment 1	Experiment 2
*N*	20 (10F)	22 (11F)
Age	71.55 (4.51)	70.27 (5.76)
Education	18.15 (7.22)	17.23 (3.15)
Shipley	37.20 (2.63)	37.09 (1.74)
MoCA	28.25 (1.12)	27.23 (1.69)*
Forced choice *d*′	1.51 (.64)	1.53 (.41)
Yes/No *d*′	1.31 (.65)	1.16 (.43)
*Note.* Standard deviations are indicated in parentheses next to mean values. Asterisks indicate a difference in scores between in the two groups (* *p* < .05). MoCA = Montreal Cognitive Assessment; Shipley = Vocabulary subtest of the Shipley Institute of Living Scale.		

**Table 5 tbl5:** Characteristics of Older Adults as a Function of Neuropsychological Group

	Recall ability group		Executive function group		Representational quality group	
Characteristic	High	Low	High	Low	High	Low
*N*	21	21	21	21	21	21
Age	68.2 (4.2)	73.6 (4.7)***	68.9 (5.3)	72.9 (4.3)*	70.8 (4.4)	71.0 (6.0)
Education	18.6 (3.2)	16.7 (7.0)	19.1 (6.4)	16.2 (3.8)	17.3 (3.3)	18.0 (7.0)
Shipley	38.0 (1.3)	36.3 (2.6)**	37.8 (1.4)	36.5 (2.6)	37.3 (2.3)	37.0 (2.1)
MoCA	28.1 (1.3)	27.3 (1.7)	28 (1.3)	27.5 (1.7)	28 (1.1)	27.5 (1.9)
Recall ability	.54 (.42)	−.54 (.43)***	.26 (.60)	−.26 (.70)*	.11 (.59)	−.11 (.78)
Executive function	.31 (.62)	−.31 (.79)**	.56 (.45)	−.56 (.60)***	.12 (.77)	−.12 (.76)
Rep quality	.43 (.76)	−.43 (1.0)**	.14 (1.0)	−.14 (1.0)	.75 (.42)	−.75 (.83)***
Forced choice *d*′	1.85 (.45)	1.19 (.40)***	1.78 (.42)	1.26 (.53)**	1.67 (.48)	1.37 (.46)*
Yes/No *d*′	1.53 (.49)	.94 (.47)***	1.46 (.52)	1.02 (.52)**	1.28 (.59)	1.20 (.54)
*Note.* Standard deviations are indicated in parentheses next to mean values. Asterisks indicate a difference between low and high scoring groups (* *p* < .05, ** *p* < .10, *** *p* < .001). MoCA = Montreal Cognitive Assessment; Shipley = Vocabulary subtest of the Shipley Institute of Living Scale.
